# MiR-519d-3p Suppresses Invasion and Migration of Trophoblast Cells via Targeting MMP-2

**DOI:** 10.1371/journal.pone.0120321

**Published:** 2015-03-24

**Authors:** Jie Ding, Fei Huang, Gaoyi Wu, Tao Han, Fuqiang Xu, Dan Weng, Chengli Wu, Xiaodong Zhang, Yuanqing Yao, Xiaoming Zhu

**Affiliations:** 1 Department of Obstetrics and Gynecology, Tangdu Hospital, The Fourth Military Medical University, Xi’an 710038, China; 2 The 307th Hospital of Chinese People’s Liberation Army, the affiliated Hospital of Military Medical Science, Beijing 100071, China; 3 Department of Stomatology, PLA Navy General Hospital, Beijing 100048, China; 4 Department of Stomatology, Jinan General Military Hospital, Jinan 250000, China; 5 Department of Orthopedics, Hainan Branch of PLA General Hospital, Hainan 572013, China; 6 Department of Gynecology and Obstetrics, Shijingshan Hospital, Beijing 100043, China; 7 Department of Cancer Research, Key Laboratory of Molecular Microbiology and Technology of Ministry of Education, Institute for Molecular Biology, College of Life Sciences, Nankai University, Tianjin 300071, China; 8 Department of Obstetrics and Gynecology, PLA General Hospital, Beijing 100853, China; Northwestern University Feinberg School of Medicine, UNITED STATES

## Abstract

Our study was approved by the Medical Ethics Committee of Tang Du Hospital, Fourth Military Medical University and complied strictly with national ethical guidelines. Preeclampsia (PE) is a specific clinical disorder characterized by gestational hypertension and proteinuria and is a leading cause of maternal and perinatal mortality worldwide. The miR-519d-3p is upregulated in the maternal plasma of patients with PE which indicates a possible association between this microRNA and the pathogenesis of PE. No studies to date have addressed the effect of miR-519d-3p on the invasion and migration of trophoblast cells. In our study, we found that miR-519d-3p expression was elevated in placental samples from patients with PE. *In vitro*, overexpression of miR-519d-3p significantly inhibited trophoblast cell migration and invasion, whereas transfection of a miR-519d-3p inhibitor enhanced trophoblast cell migration and invasion. Luciferase assays confirmed that matrix metalloproteinase-2 (MMP-2) is a direct target of miR-519d-3p. Quantitative real-time PCR and western blot assays showed that overexpression of miR-519d-3p downregulated MMP-2 mRNA and protein expression. Knockdown of *MMP-2* using a siRNA attenuated the increased trophoblast migration and invasion promoted by the miR-519d-3p inhibitor. In placentas from patients with PE or normal pregnancies, a negative correlation between the expression of *MMP-2* and miR-519d-3p was observed using the Pearson correlation and linear regression analysis. Our present findings suggest that upregulation of miR-519d-3p may contribute to the development of PE by inhibiting trophoblast cell migration and invasion via targeting *MMP-2*; miR-519d-3p may represent a potential predictive and therapeutic target for PE.

## Introduction

Preeclampsia (PE) is a specific disorder characterized by gestational hypertension and proteinuria as the main clinical symptoms [1]. Although the exact pathophysiological mechanism leading to PE remains uncertain, it is generally considered that PE is a multifactorial disorder with placental oxygen disruption [2], inappropriate maternal vascular damage [3], anomalous maternal-fetal immune interactions [4,5], abnormal trophoblast cell invasion [6], and other processes involved. Recent reports have shown that matrix metalloproteinase-2 (MMP-2), an important zinc-dependent protease in the MMP superfamily that breaks down extracellular matrix components [7], may be involved in cytotrophoblastic invasion during embryogenesis. Dysregulation of MMP-2 expression and impaired MMP-2 activity are involved in abnormal uteroplacental artery remodeling and trophoblastic invasion in hypertension of pregnancy [8]. Downregulation of MMP-2, in conjunction with inflammation and oxidative factors, causes trophoblastic cell dysfunction in PE [9,10]; however, the underlying molecular mechanism remains unclear.

MicroRNAs (miRNAs) are a class of endogenous, non-coding, small RNAs around 20 to 25 nucleotides long that act as important negative post-transcriptional regulators of gene expression [11–13]. Recently, several studies have used miRNA microarray approaches to identify differentially expressed miRNAs in placentas from patients with normal pregnancies and those with PE [11–14]. The results have revealed numerous differently expressed miRNAs, such as aberrant overexpression of miR-29b [15], miR-16 [16] and miR-222 [17], may play essential roles during the pathogenesis of PE [18]. However, the potential effects and mechanisms by which miRNAs regulate trophoblastic cell function are poorly characterized and need to be investigated further.

MiR-519d-3p can inhibit cell growth by targeting MKi67 to induce DNA hypomethylation in HCC [19–21]. MiR-519d-3p could repress ovarian cancer cell proliferation and promote cell death by targeting X-linked inhibitor of apoptosis protein (XIAP) [19]. The studies revealed that miR-519d-3p is upregulated in the maternal plasma of patients with PE [19–22]. Bioinformatic analysis using miRanda, TargetScan and miRBase indicated that MMP-2 is a commonly-predicted target gene of miR-519d-3p. However, there has been no study addressing the impact of miR-519d-3p on trophoblast cell invasion and migration.

In our study, we hypothesized that miR-519d-3p may participate in the regulation of trophoblast cell invasion and migration by targeting MMP-2. We compared the expression of miR-519d in placentas from patients with normal pregnancies and those with PE, as well as a normal trophoblast cell line, HTR8/Svneo, and trophoblast tumor cell line, JEG-3. We investigated the ability of miR-519d-3p to regulate trophoblast cell invasion and migration, confirmed the predicted binding site for miR-519d-3p in the MMP-2 3`untranslated region (UTR), determined the effect of MMP-2 on miR-519d-3p-regulated trophoblast cell invasion and migration, and analyzed the correlation between the expression of miR-519d-3p and MMP-2 in placentas from patients with PE. The findings of our study confirm that miR-519d-3p can regulate trophoblast cell invasion and migration by targeting MMP-2, and indicate that miR-519d-3p may participate in the pathological processes underlying PE.

## Materials and Methods

### Ethics statement

Our study was approved by the institutional review board of the Fourth Military Medical University. All participants provide their written consent to participate in our study. Additionally, the Ethics Committee of Fourth Military Medical University approved the use of the obtained patient data for our study.

### Clinical specimen collection

Specimen classification and the inclusion criteria were as follows: normal pregnancy was defined as patients with no history of hypertension or proteinuria during weeks 35–40 of pregnancy who delivered healthily neonates via cesarean section. Severe PE was strictly defined according to the International Society for the Study of Hypertension in Pregnancy [23]. Patients with severe PE were puerperas with no history of chronic hypertension or proteinuria, but who experienced severe hypertension (systolic blood pressure ≥ 160 mmHG and/or diastolic blood pressure ≥ 110 mmHG) plus severe proteinuria (≥ 2.0 g per 24 h or greater than 2+ by dipstick) during pregnancy. Puerperas who had renal disease, hypertension before pregnancy, placental abruption or placenta praevia, anemia or other hematologic disease, gestational diabetes, fetal distress syndrome or growth retardation were excluded from our study. Placental tissues from 21 women with normal pregnancies and 18 patients with severe PE were collected after cesarean section at the Department of Obstetrics and Gynaecology, Tangdu hospital, 2nd Affiliated Hospital of Forth Military Medical University, China. All of the patients in our study were primiparas and the general clinical data, such as age, gestational week, infant birth weight, etc., were matched between groups (Table 1).

**Table 1:** Clinical parameters of patients enrolled in our study.

	Normal (n = 21)	PE (n = 18)	P-Value
Age (y)	30.05 ± 0.72	28.44 ± 0.95	0.1799
SBP (mmHg)	116.0 ± 1.91	169.8 ± 1.41	<0.0001
DBP (mmHg)	70.71±1.43	104.4 ± 2.54	<0.0001
Proteinuria (g/24h)	NA	3.58 ± 0.26	NA
Nulliparous (%)	90.5	88.9	NA
Gestational day at delivery (Day)	272.9 ± 3.08	252.1 ± 2.98	<0.0001
Infant birth weight (g)	3119 ± 123.4	2344 ± 90.62	<0.0001

Data are shown as Mean ± SEM, and significant difference between Normal and PE patients are analyzed with Student’s *t* test. SBP, systolic blood pressure; DBP, diastolic blood pressure; NA, not available.

### Cell culture and transfection

The human immortalized trophoblast cell line HTR8/SVneo and human trophoblast tumor cell line JEG-3 were cultured in DMEM-F12 medium (Hyclone, Logan, USA) supplemented with 10% fetal bovine serum (Gibco, Carlsbad, CA, USA) in a 37°C humidified incubator (5% CO2), and subcultured at ratio of 1:3 when the cells reached 80–90% confluence.

For transient transfection, HTR8/SVneo cells were seeded in complete serum medium the day before transfection, then transfected with miR-519d-3p mimics or inhibitor using Lipofectamine 2000 Reagent (Invitrogen, Carlsbad, CA, USA) in Opti-MEM (Hyclone, Logan, USA). The transfection medium was replaced with complete medium 4 h after transfection, and the cells were harvested for subsequent experimental studies 24 h after transfection.

### RNA extraction and quantitative real-time PCR

Total RNA was extracted from clinical samples and cells using TRIzol reagent (Invitrogen, Carlsbad, CA, USA) following the manufacturer’s instructions. Reverse transcription of mRNA and miRNAs were performed using the PrimeScript RT Master Mix and SYBR PrimeScript miRNA RT-PCR Kits, respectively (Takara Biotechnology, Dalian, China). Quantitative real-time PCR was performed using SYBR Premix Ex Taq (Tli RNaseH Plus; Takara Biotechnology, Dalian, China) using the Applied Biosystems 7500 (Life Technologies); β-actin or U6 were used as internal controls to normalize the relative expression level of the target genes and miRNAs, respectively.

### Protein extraction and Western blotting

Total cellular proteins were extracted, subjected to SDS-page electrophoresis and transferred to PVDF membranes using standard procedures. The primary antibodies included rabbit polyclonal anti-MMP-2 (1:1000; Abcam(Hong Kong) Ltd, HK, China) and mouse monoclonal anti-β-actin (1:1000; Sigma, St. Louis, USA); β-actin was used as an internal loading control. The bands were visualized and imaged using eECL Western Blot Kit (Cwbiotech, Beijing, China) and densitometry was performed using Image J (Version 1.49e, NIH, USA).

### Transwell invasion assay

Cell invasion was assessed using Transwell chambers (24-well inserts; 8 um-pore size; Millipore, Billerica, MA, USA) that had been pre-coated with Matrigel (200 μg/ml, BD Biosciences, Franklin Lakes, New Jersey, USA) as previously described [19]. Briefly, 24 h after transfection, the cells were treated with 10 μg/ml mitomycin C for 2 h, trypsinized and seeded into the upper chambers (1 × 10^5^ cells/chamber) in serum-free medium, and the lower chambers were filled with DMEM-F12 medium containing 15% FBS as a chemoattractant. The plates were incubated for 48 h, then the cells on the upper surface of the chambers were removed gently and the invaded cells on the surface of the lower chambers were fixed in methanol, stained with crystal violet, and the number of cells in four randomly-selected fields of view were imaged using a light microscope and counted.

### Wound healing assay

Cells were seeded into 24-well plates, transfected with the miRNA or siRNA (or controls) and cultured for 24 h until about 70% confluent. Prior to the wound healing assay, the cells were treated with 10 μg/ml mitomycin C for 2 h to suppress proliferation, then a wound was created in the monolayer using a sterilized 200 μl pipette tip, and the width of each wound was measured using Image J at 0 h, 24 h and 48 h after wounding. Cell migration was expressed by subtracting the wound width at 24 h or 48 h from that at 0 h.

### Luciferase reporter assay

The nucleotides encoding 867–1183 nt of the 3`UTR of human MMP-2 mRNA (Genbank accession no. NM_001127891.2) that contain the predicted miR-519d-3p target site (1149–1171) were amplifed by PCR from HeLa cell cDNA and then cloned into the pGL3-promoter vector (Promega BioSciences, San Luis Obispo, CA, USA); this plasmid was named pGL3-MMP2. The pGL3-MMP2 mutant plasmid containing eight single nucleotide mutations (1163–1170) in the predicted MMP-2 3`UTR binding site was constructed based on pGL3-MMP2 by PCR mutagenesis according the manufacture’s instruction (Stratagene, La Jolla, California, USA). HTR8/SVneo cells were seeded at 1.0 × 10^5^ per well in 24-well plates, cultured until 70% confluent and co-transfected with miR-con (scramble, 50 nM; Ribobio Biotechnology, Guangzhou, China) or miR-519d-3p mimic (50 nM, Ribobio Biotechnology); pGL3-control (400 ng; Promega), pGL3-MMP2 (400 ng) or pGL3-MMP2 mutant; and pRL-TK (50 ng, Promega) using Lipofectamine 2000. Cells were lysed using cell lysis buffer (Cell Signaling, Boston, MA, USA) and luciferase activity was measured using the Dual-Luciferase Reporter Assay System (Promega BioSciences, San Luis Obispo, CA, USA) according to the manufacturer’s instructions.

### Statistical analysis

GraphPad InStat software (Graphpad, San Diego, USA) was used for statistical analysis. Data from the quantitative real-time PCR, Western blot, Transwell invasion, wound healing and luciferase reporter assays are presented as the mean ± SEM values of three independent experiments. Group comparisons were performed using the Student’s *t* test. The correlation between miR-519d-3p and MMP-2 expression in placentas from patients with PE were analyzed using the Pearson correlation and linear regression analysis. Statistical significance was defined as *P* < 0.05.

## Results

### MiR-519d-3p is upregulated in the placenta of patients with PE and downregulated in a trophoblastic tumor cell line

**Fig 1:**
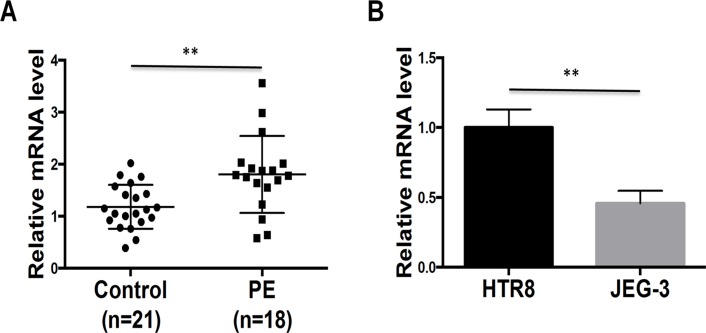
Expression of miR-519d-3p is upregulated in the placentas of patients with preeclampsia and a trophoblast cell line. Quantitative real-time PCR analysis of miR-519d expression in placentas from patients with severe preeclampsia (PE) and matched normal pregnancies (Control) (A), and the normal trophoblast cell line HTR8 and trophoblastic tumor cell line JEG-3 (B). Relative expression of miR-519d was normalized to U6. *P < 0.05, **P < 0.01, *** *P* < 0.001.

To examine the role of miR-519d-3p in trophoblast cells, we first quantified the expression of miR-519d-3p in placental tissues from 21 women with normal pregnancies and 18 patients with severe PE by qRT-PCR. As shown in Fig. 1A, the expression of miR-519d-3p was generally higher in placental tissues from patients with PE than those with normal placental tissues. Subsequently, we examined the expression of miR-519d-3p by qRT-PCR in the normal trophoblast cell line HTR8/SVneo and trophoblastic tumor cell line JEG-3. As shown in Fig. 1B, miR-519d-3p was significantly downregulated in JEG-3 cells compared to HTR8/SVneo cells. These data indicate that the expression of miR-519d-3p may be closely related to trophoblast cell function.

### MiR-519d-3p suppresses the invasion and migration of trophoblast cells

**Fig 2:**
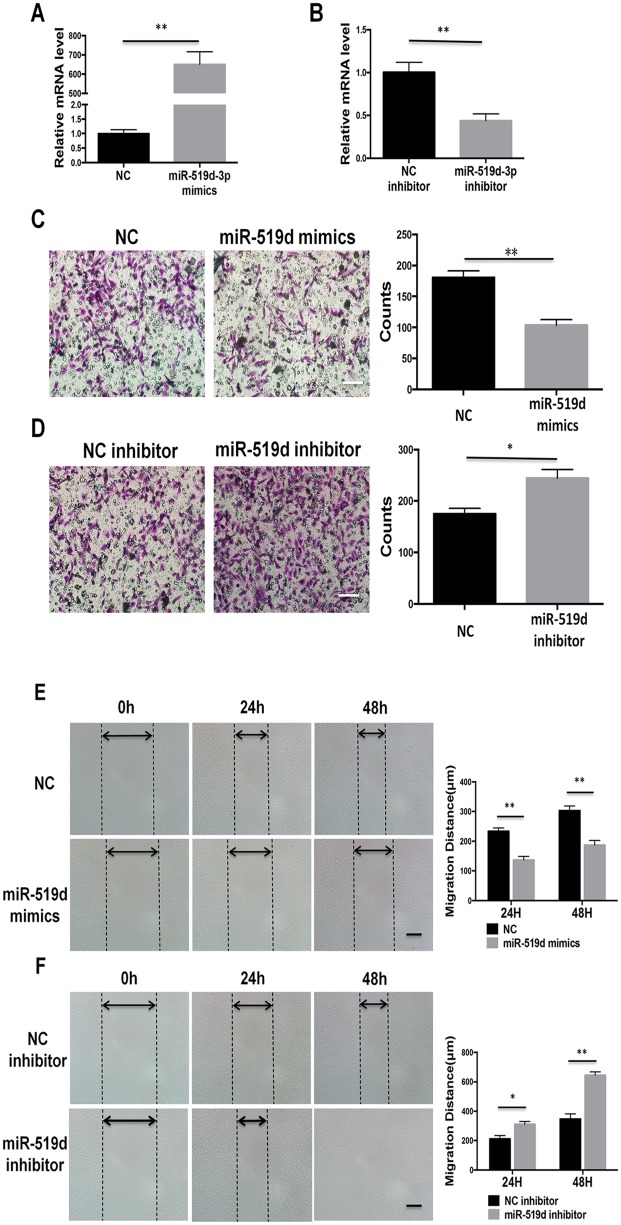
MiR-519d-3p suppresses trophoblast cell invasion and migration. (A, B) Quantitative real-time PCR analysis of miR-519d-3p expression in HTR8/SVneo cells transfected with miR-519d-3p mimic (A), miR-519d-3p inhibitor (B) or the corresponding controls (NC or NC inhibitor). Relative expression of miR-519d-3p was normalized to U6. (C, D) Transwell invasion assay of HTR8/SVneo cells transfected with miR-519d-3p mimic (C), miR-519d-3p inhibitor (D) or the corresponding controls (NC or NC inhibitor). (E, F) Wound healing assay of HTR8/SVneo cells transfected with miR-519d-3p mimic (E), miR-519d-3p inhibitor (F) or the corresponding controls (NC or NC inhibitor). Scale bar refers to 200um, * *P* < 0.05.

The Transwell invasion assay was used to evaluate whether miR-519d-3p affects the invasive ability of trophoblast cells. As shown in Fig. 2A and 2B, transfection of miR-519d mimic and miR-519d inhibitor significantly increased and decreased, respectively, the relative expression of miR-519d-3p in HTR8/SVneo cells. As shown in Fig. 2C and 2D, overexpression of miR-519d-3p significantly inhibited HTR8/SVneo cell invasion at 48 h after transfection with the miR-519d-3p mimic compared to the miR-control group. Downregulation of miR-519d-3p by transfection of the miR-519d-3p inhibitor significantly increased the invasive ability of HTR8/SVneo cells. The wound healing assay was used to evaluate the influence of miR-519d-3p on trophoblast cell migration. As shown in Fig. 2E and 2F, overexpression of miR-519d-3p significantly suppressed HTR8/SVneo cell migration at 24 h and 48 h after transfection with miR-519d-3p mimic, whereas inhibition of miR-519d-3p promoted HTR8/SVneo cell migration at 24 h and 48 h after transfection of the miR-519d-3p inhibitor. These data indicate that miR-519d-3p suppresses trophoblast cell invasion and migration.

### MMP-2 is a direct target of miR-519d-3p

**Fig 3:**
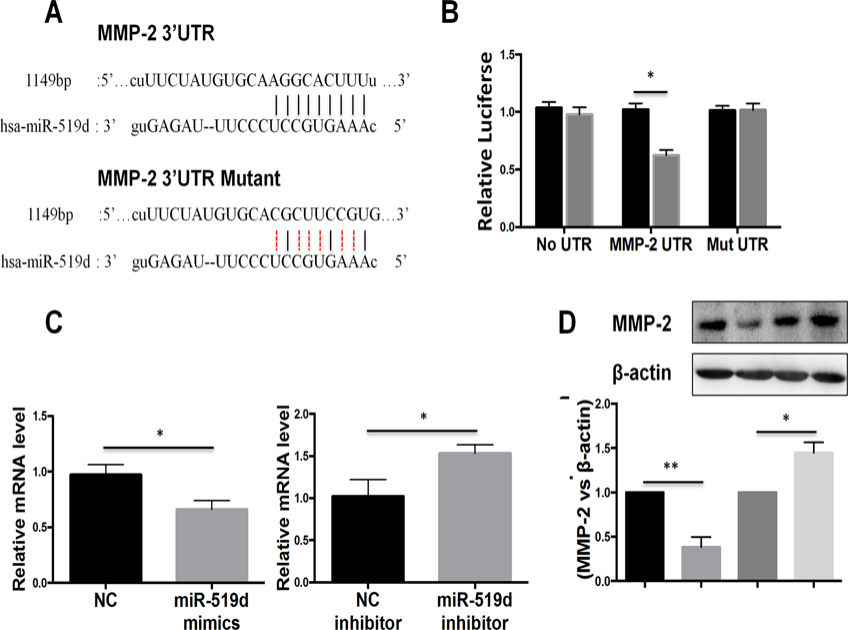
MMP-2 is a direct target of miR-519d-3p. (A) Schematic illustrating construction of the luciferase reporter constructs (MMP-2 UTR and MMP-2 Mut UTR). (B) Luciferase assay of HTR8/SVneo cells co-transfected with miR-519d-3p mimics (or negative control) and the wild-type MMP-2 3`UTR reporter construct (MMP-2 UTR) or mutated MMP-2 3`UTR reporter construct (Mut UTR). (C, D) Quantitative real-time PCR and Western blot analysis of endogenous MMP-2 mRNA (C) and protein (D) expression in HTR8/SVneo cells transfected with miR-519d-3p mimic or miR-519d-3p inhibitor; data is expressed relative to the corresponding control cells (NC or NC inhibitor), *P* < 0.05.

To investigate the mechanism by which miR-519d-3p regulates the invasion and migration of trophoblast cells, we performed bioinformatic analysis of miR-519d-3p. The bioinformatic analysis revealed a putative miR-519d-3p binding site in 3`UTR of MMP-2 (Fig. 3A); this gene is closely associated with the invasion and migration of trophoblast cells [8]. To confirm that miR-519d-3p directly targets MMP-2, we performed luciferase reporter assays in trophoblast cells. As shown in Fig. 3B, the luciferase activity of the wild-type MMP-2 3`UTR reporter gene was markedly lower in cells transfected with miR-519d-3p mimic compared to negative control miRNA transfected-cells; however, this reduction in luciferase activity was abolished by mutation of the putative miR-519d-3p binding site in the MMP-2 3`UTR reporter gene. To further validate the association between miR-519d-3p and MMP-2, we quantified endogenous MMP-2 mRNA expression in HTR8/SVneo cells transfected with miR-519d-3p mimic or miR-519d-3p inhibitor. Quantitative RT-PCR demonstrated that transfection of the miR-519d-3p mimic significantly reduced the expression of endogenous MMP-2 mRNA, whereas the miR-519d-3p inhibitor significantly increased MMP-2 mRNA expression (Fig. 3C). Western blotting confirmed that the expression of MMP-2 significantly reduced in cells transfected with miR-519d-3p mimic and increased in cells transfected with the miR-519d-3p inhibitor (Fig. 3D). Taken together, these results indicate that MMP-2 is a direct target gene of miR-519d-3p.

### Restoring MMP-2 expression reverses the regulation of miR-519d-3p on trophoblast cell invasion and migration

**Fig 4:**
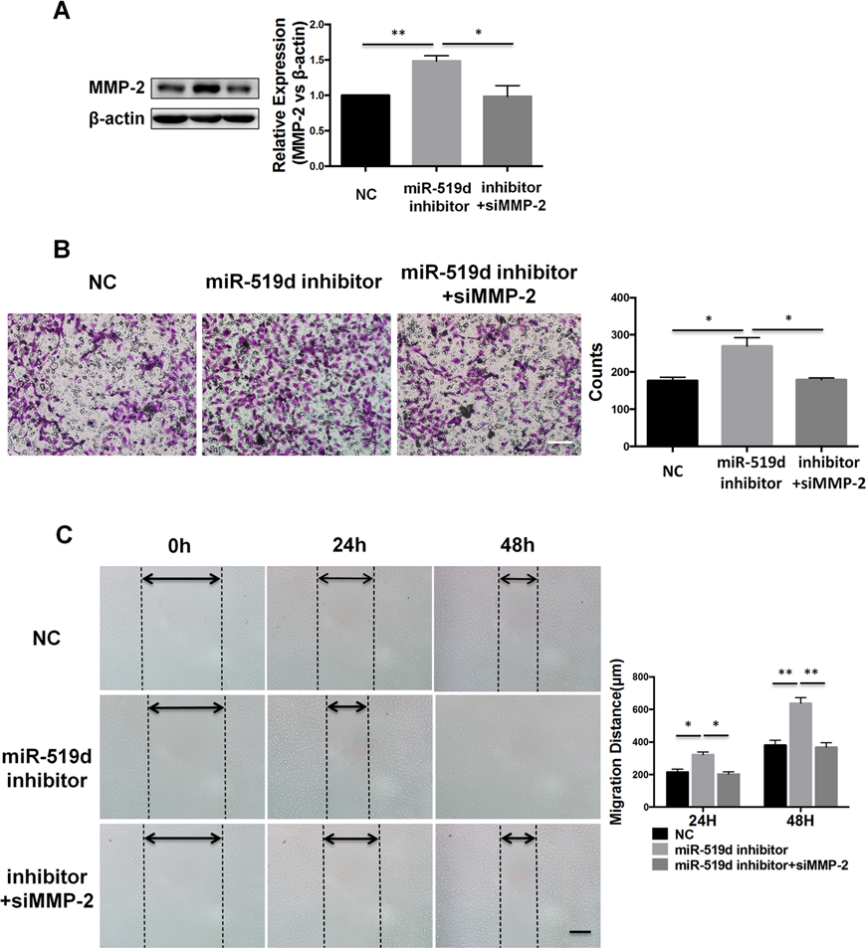
Restoring MMP-2 expression reverses the regulation of miR-519d-3p on trophoblast cell invasion and migration. HTR8/SVneo cells were transfected with miR-519d-3p inhibitor alone, miR-519d-3p inhibitor plus MMP-2 siRNA, and the corresponding control (NC) (A) Western blot assay of MMP-2 protein expression. (B) Transwell invasion assay. (C) Wound healing assay. Scale bar refers to 200um. * *P* < 0.05.

MMP-2 has been reported to play a critical role in the migration and invasion of normal trophoblast cells and various trophoblastic cancers [8]. To verify whether the function of miR-519d-3p is exerted via regulation of MMP-2, we co-transfected HTR8/SVneo cells with the miR-519d-3p inhibitor and MMP-2 siRNA to perform a rescue experiment. As shown in Fig. 4A, Western blot analysis showed that simultaneous transfection of HTR8/SVneo cells with the miR-519d-3p inhibitor and MMP-2 siRNA reduced the ability of the miR-519d-3p inhibitor to upregulate MMP-2 protein expression. Furthermore, the Transwell invasion assay revealed that co-transfection of MMP-2 siRNA significantly reduced the ability of the miR-519d-3p inhibitor to promote cell invasion at 48 h after transfection (Fig. 4B). Additionally, the wound-healing assay showed that co-transfection of MMP-2 siRNA abolished the increased cell migration promoted by transfection of the miR-519d-3p inhibitor at 24 h and 48 h (Fig. 4C). These results indicate that miR-519d-3p suppresses trophoblast cell invasion and migration via regulating MMP-2.

### Expression of MMP-2 correlates negatively with miR-519d-3p in placental tissues

**Fig 5:**
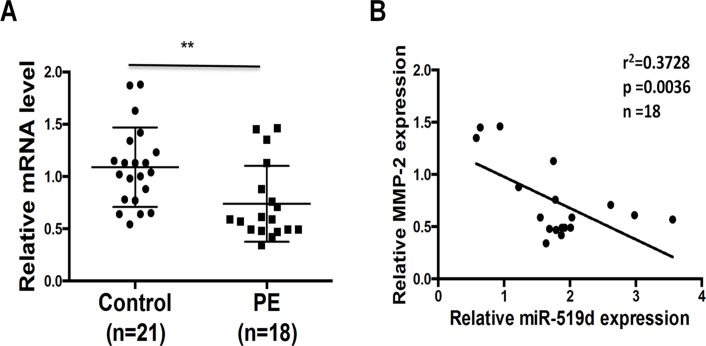
Expression of MMP-2 correlates negatively with miR-519d-3p in placental tissues. (A) Quantitative real-time PCR analysis of MMP-2 mRNA expression in placentas from patients with PE (PE) and normal pregnancies (control). Relative expression of MMP-2 was normalized to β-actin. Data is mean ± SEM of three independent experiments; *, *P* < 0.05 vs. control, Student’s *t*-test. (B) The correlation between miR-519d-3p and MMP-2 expression in placentas from patients with PE was analyzed using the Pearson correlation and linear regression analysis. Relative expression of miR-519d and MMP-2 were normalized to U6 or β-actin, respectively.

In order to further elucidate the correlation between MMP-2 and miR-519d-3p, we used qRT-PCR to quantify the expression of MMP-2 and miR-519d-3p in the placentas of patients with PE. As shown in Fig. 5A and 5B, the expression of MMP-2 mRNA was reduced in the placentas of patients with PE compared to patients with normal pregnancies, and the expression of MMP-2 correlated negatively with the expression of miR-519d-3p. These results indicate that miR-519d-3p can negatively regulate the expression of MMP-2 *in vivo*, indicating that miR-519d-3p may participate in the pathological processes underlying PE.

## Discussion

In our study, we found that miR-519d-3p was overexpressed in the placentas of patients with PE, and downregulated in a trophoblast tumor cell line compared to a normal trophoblast cell line. We also proved that miR-519d-3p could suppress the invasion and migration of trophoblast cells via targeting MMP-2 using in vitro functional and rescue experiments. Furthermore, we demonstrated an inverse correlation exists between the expression of miR-519d-3p and MMP-2 in the placentas of patients with PE.

Recent research indicated reported that miRNA expression profiles were altered in the placentas of patients with PE compared to normal placentas [18,24,25]. MiR-378a-5p restrains trophoblast cell functions [26]. MiR-29b participates in the onset of PE by repressing trophoblast cell invasion and angiogenesis and enhancing cell apoptosis through targeting MMP-2, Integrin beta 1 (ITGB1) and vascular endothelial growth factor A (VEGF-A) [15]. In our study, we observed that the expression of miR-519d was significantly upregulated in the placentas of patients with PE compared to those of normal pregnancies, and the expression of miR-519d was downregulated in a trophoblast tumor cell line compared to a normal trophoblast cell line. MiR-519d-3p is a member of the chromosome 19 miRNA cluster (C19MC), which is exclusively expressed in the placenta and functions as an important regulator of placental–maternal signaling [27]. A recent study showed that circulating C19MC miRNAs play important roles in the pathogenesis of PE, and upregulation of C19MC miRNAs, such as miR-516–5p, miR-517* and miR-526a, could be used as a biomarker to diagnose PE [28,29]. To date, it has been reported that miR-519d-3p promotes cell proliferation and metastasis in a range of pathological processes such as obesity [30], osteosarcoma [31], hepatocellular carcinoma [20,21] and ovarian cancer [19]; however, a role for miR-519d in PE had not been reported.

It is generally accepted that shallow invasion of trophoblast cells into the decidua and subsequent defects in spiral artery remodeling are involved in the onset of PE [1]. In order to study the function of miR-519d-3p, we examined the effects of miR-519d on the migration and invasion of trophoblast cells in vitro by transiently transfecting a trophoblast cell line, HTR8/SVneo, with a miR-519d-3p mimic or miR-519d-3p inhibitor. Overexpression of miR-519d-3p potently suppressed trophoblast cell migration and invasion in the wound healing and Transwell invasion assays. In addition, inhibition of endogenous miR-519d-3p significantly promoted trophoblast cell migration and invasion. Combined with our findings that the expression of miR-519d-3p is upregulated in the placentas of patients with PE compared to those of normal pregnancies, these observations indicate that miR-519d-3p may play an important role in abnormal trophoblast migration and invasion during the pathological processes leading to PE.

In our study, we demonstrated that miR-519d-3p interacted with its partially complementary sequence in the 3`UTR of MMP-2 using luciferase reporter assays, and confirmed that miR-519d-3p negatively regulated MMP-2 mRNA and protein expression using qRT-PCR and Western blot analyses. In functional rescue experiments, we found that knockdown of MMP-2 using a siRNA suppressed trophoblast cell migration and invasion; in a similar manner as overexpression of miR-519d-3p. Additionally, the MMP-2 siRNA reversed the ability of miR-519d to promote trophoblast cell migration and invasion. Furthermore, to confirm the regulation of MMP-2 by miR-519d-3p in vivo, we analyzed the expression of miR-519d-3p and MMP-2 in the placentas of patients with PE and those of patients with normal pregnancies, and observed an inverse correlation between the expression of MMP-2 and miR-519d-3p. Consistent with our previous observations described above, these findings demonstrate that miR-519d-3p regulates trophoblast cell migration and invasion by targeting MMP-2.

In summary, our study extends our knowledge of the mechanism of action of miRNAs during the pathogenesis of PE. Upregulation of miR-519d-3p may contribute to the occurrence of PE by inhibiting trophoblast cell migration and invasion via targeting MMP-2, suggesting miR-519d-3p may have potential as a predictive or therapeutic target for PE.
